# Supplementary material for: Learning produces an orthogonalized state machine in the hippocampus
Source: Nature. 2025 Feb 12;640(8057):165–75. doi: 10.1038/s41586-024-08548-w (PMC11964937; doi:10.1038/s41586-024-08548-w)
Supplement: Supplementary file 1 — Reporting Summary [file 41586_2024_8548_MOESM1_ESM.pdf]

nature portfolio

Corresponding author(s): Nelson Spruston, Weinan Sun

Last updated by author(s): Nov 4, 2024

Reporting Summary

Nature Portfolio wishes to improve the reproducibility of the work that we publish. This form provides structure for consistency and transparency in reporting. For further information on Nature Portfolio policies, see our Editorial Policies and the Editorial Policy Checklist.

Statistics

For all statistical analyses, confirm that the following items are present in the figure legend, table legend, main text, or Methods section.

n/a	Confirmed	
☐	☒	The exact sample size (*n*) for each experimental group/condition, given as a discrete number and unit of measurement
☐	☒	A statement on whether measurements were taken from distinct samples or whether the same sample was measured repeatedly
☐	☒	The statistical test(s) used AND whether they are one- or two-sided<br>*Only common tests should be described solely by name; describe more complex techniques in the Methods section.*
☒	☐	A description of all covariates tested
☒	☐	A description of any assumptions or corrections, such as tests of normality and adjustment for multiple comparisons
☐	☒	A full description of the statistical parameters including central tendency (e.g. means) or other basic estimates (e.g. regression coefficient) AND variation (e.g. standard deviation) or associated estimates of uncertainty (e.g. confidence intervals)
☐	☒	For null hypothesis testing, the test statistic (e.g. *F*, *t*, *r*) with confidence intervals, effect sizes, degrees of freedom and *P* value noted<br>*Give P values as exact values whenever suitable.*
☒	☐	For Bayesian analysis, information on the choice of priors and Markov chain Monte Carlo settings
☒	☐	For hierarchical and complex designs, identification of the appropriate level for tests and full reporting of outcomes
☐	☒	Estimates of effect sizes (e.g. Cohen's *d*, Pearson's *r*), indicating how they were calculated

Our web collection on statistics for biologists contains articles on many of the points above.

Software and code

Policy information about availability of computer code

Data collection	ScanImage® 2021.0.0, Matlab R2021a
Data analysis	Python 3.8, Suite2p 0.10.3, Matlab R2021a, and custom code for VR logging: https://github.com/winnubstj/Gimbl and generating the figures: https://github.com/sprustonlab/OSM_Paper_Figures

For manuscripts utilizing custom algorithms or software that are central to the research but not yet described in published literature, software must be made available to editors and reviewers. We strongly encourage code deposition in a community repository (e.g. GitHub). See the Nature Portfolio guidelines for submitting code & software for further information.

Data

Policy information about availability of data

All manuscripts must include a data availability statement. This statement should provide the following information, where applicable:

- Accession codes, unique identifiers, or web links for publicly available datasets
- A description of any restrictions on data availability
- For clinical datasets or third party data, please ensure that the statement adheres to our policy

Imaging data are available at Figshare: https://doi.org/10.25378/janelia.2727355267. We also provide an interactive data visualization tool at http://cognitivemap.janelia.org.

Research involving human participants, their data, or biological material

Policy information about studies with human participants or human data. See also policy information about sex, gender (identity/presentation), and sexual orientation and race, ethnicity and racism.

Reporting on sex and gender	N/A
Reporting on race, ethnicity, or other socially relevant groupings	N/A
Population characteristics	N/A
Recruitment	N/A
Ethics oversight	N/A

Note that full information on the approval of the study protocol must also be provided in the manuscript.

Field-specific reporting

Please select the one below that is the best fit for your research. If you are not sure, read the appropriate sections before making your selection.

☒ Life sciences ☐ Behavioural & social sciences ☐ Ecological, evolutionary & environmental sciences

For a reference copy of the document with all sections, see nature.com/documents/nr-reporting-summary-flat.pdf

Life sciences study design

All studies must disclose on these points even when the disclosure is negative.

Sample size	Sample sizes were determined based on previous studies investigating hippocampal neural activity during learning in head-fixed mice. No explicit power analysis was performed, but the number of animals and recorded neurons was comparable to or exceeded those used in similar published work in the field.
Data exclusions	4 mice that did not reach expert performance within 15 days of task training were excluded from analysis. Imaging for 5 mice with damaged or degraded imaging window were terminated and the data were excluded from the study.
Replication	The experiments were independently replicated across four different groups mice by five different team members (including 3 postdocs, two technicians). Each experimenter independently trained and tested their own mouse/mice on the task. While individual animals showed some behavioral variations, the key qualitative findings were consistent across all cohorts and experimenters. The data analysis procedures and main findings were independently validated by at least five co-authors, each replicating the key results.
Randomization	Our study was observational and focused on tracking the progression of neural activity over time in a group of animals. Since there were no interventions or treatment groups to assign, and all subjects underwent the same imaging process, randomization was not necessary or applicable for our research objectives.
Blinding	Blinding was not employed in this observational study as it involved direct imaging of neural activity, where observer bias is unlikely and blinding is not typically applicable.

Reporting for specific materials, systems and methods

We require information from authors about some types of materials, experimental systems and methods used in many studies. Here, indicate whether each material, system or method listed is relevant to your study. If you are not sure if a list item applies to your research, read the appropriate section before selecting a response.

Materials & experimental systems

n/a	Involved in the study	
☒	☐	Antibodies
☒	☐	Eukaryotic cell lines
☒	☐	Palaeontology and archaeology
☐	☒	Animals and other organisms
☒	☐	Clinical data
☒	☐	Dual use research of concern
☒	☐	Plants

Methods

n/a	Involved in the study	
☒	☐	ChIP-seq
☒	☐	Flow cytometry
☒	☐	MRI-based neuroimaging

Animals and other research organisms

Policy information about studies involving animals; ARRIVE guidelines recommended for reporting animal research, and Sex and Gender in Research

Laboratory animals	Thy1-GCaMP6f transgenic mice (JAX line GP5.17) were used in this study, aged 3-6 months at the time of surgery (3-8 months at the beginning of imaging studies). Mice were housed in a temperature and humidity-controlled vivarium maintained at 21 ± 1°C and 50 ± 10% relative humidity, with a reversed 12-hour light-dark cycle.
Wild animals	This study did not involve wild animals.
Reporting on sex	Mice of both sexes were included in the study.
Field-collected samples	No field collected samples were used in the study.
Ethics oversight	Janelia Research Campus Institutional Animal Care and Use Committee.

Note that full information on the approval of the study protocol must also be provided in the manuscript.

Plants

Seed stocks	*Report on the source of all seed stocks or other plant material used. If applicable, state the seed stock centre and catalogue number. If plant specimens were collected from the field, describe the collection location, date and sampling procedures.*
Novel plant genotypes	*Describe the methods by which all novel plant genotypes were produced. This includes those generated by transgenic approaches, gene editing, chemical/radiation-based mutagenesis and hybridization. For transgenic lines, describe the transformation method, the number of independent lines analyzed and the generation upon which experiments were performed. For gene-edited lines, describe the editor used, the endogenous sequence targeted for editing, the targeting guide RNA sequence (if applicable) and how the editor was applied.*
Authentication	*Describe any authentication procedures for each seed stock used or novel genotype generated. Describe any experiments used to assess the effect of a mutation and, where applicable, how potential secondary effects (e.g. second site T-DNA insertions, mosiacism, off-target gene editing) were examined.*
